# Facilitators and Barriers That Transfemoral Amputees Experience in Their Everyday Life: A Norwegian Qualitative Study

**DOI:** 10.1155/2022/2256621

**Published:** 2022-11-08

**Authors:** Inger Lise Sørensen, Geir V. Berg

**Affiliations:** ^1^Department of Physical Medicine and Rehabilitation, Soerlandet Hospital, Kristiansand, Norway; ^2^Department of Health and Nursing Sciences, Inland Norway University of Applied Sciences Elverum, Elverum, Norway

## Abstract

**Background:**

Living with a lower limb amputation influences multiple facets of life due to altered function. Individuals achieve a varied level of function post amputation, depending on several variables like age, level of function prior to amputation, and available personal and environmental resources. Releasing the potential to live life to the fullest despite a disability is important to the individual.

**Objectives:**

The primary objective of this study is to identify barriers and facilitators for function which lower limb amputees experience in their lives several years after amputation, from the amputee's perspective. This knowledge can contribute to further development of the clinical pathway for lower limb amputees in a Norwegian rehabilitation hospital.

**Methods:**

The study has a descriptive and exploratory qualitative design with a phenomenological hermeneutical approach. Semistructured, individual interviews were conducted for data collection. Thematic analysis inspired by Braun and Clarke was used for data analysis. The sample consisted of eight transfemoral amputees (70 ± 6.9 (58-77 years)) living in the southern part of Norway. Average time since amputation was 11 years.

**Results:**

The results have been categorised into two main themes with subthemes: (1) facilitators: personal resources, a well-fitted prosthesis, rehabilitation, social network, balance in activity/rest, and accessibility and (2) barriers: walking distance, poorly fitted prosthesis, pain, comorbidities, climate/terrain/falling, reduced local competence on amputation, and pandemic.

**Conclusion:**

Lower limb amputees experience barriers in their everyday life, but they also develop strategies to cope with their disability. Clinical implications can include increased nutritional guidance, structural psychological mapping and follow-up, structured follow-ups over a significant period of time, and extended use of digital consultation.

## 1. Introduction

Lower limb amputation (LLA) is a surgical treatment which may be necessary in order to save lives [1] and can represent a dramatic event for the individual. An amputation will to a variable extent influence functioning in the patients' everyday life, both physically, psychologically, practically, financially, and socially [2–5]. Annual expenses for the Norwegian society are approximately 50 million US$ [6].

An LLA patient can undergo treatment in a prosthetic rehabilitation programme. This is a complex process, which includes recovering from the surgery into ambulating with a prosthesis. Major stages in the rehabilitation process can include healing of the residual limb, prevention of complications, and promotion of functional independence [7], with a lifelong perspective. The process requires a highly specialised multidisciplinary team [3, 7, 8], which preferably take a holistic approach. The purpose of rehabilitation is to optimise health, function, independence, and quality of life [8].

The clinical pathway for primary rehabilitation of major lower limb amputation at the rehabilitation unit in Soerlandet Hospital starts 5-7 days postoperatively. A prosthesis is fitted approximately three weeks post amputation, and the patients undergo training to manage the basic skills living with and without a prosthesis. On average, the primary rehabilitation phase is six weeks before the patients are discharged to their homes or to local health services. The patients come to an outpatient, multidisciplinary consultation three and twelve months post discharge. Throughout this clinical pathway, objective measures on functional outcomes and self-reported health-related quality of life (HRQOL) [9] are performed. HRQOL includes “aspects of overall quality of life that can be clearly shown to affect health—either physical or mental” [10, 11].

In the current study, we wanted to gain further in-depth knowledge on patient experience; hence, we conducted a formal qualitative study to collect data from the patient's perspective.

The research question was as follows:

*Which barriers and facilitators did lower limb amputees experience in their everyday life some years post discharge from primary rehabilitation?*

To assess how the participants cope with their disability, the results of the study were contextualised in terms of motivation. We chose to use the self-determination theory as a theoretical framework [12].

In the self-determination theory (SDT), the three terms autonomy, competence, and relatedness are central as basic human needs. Autonomy means performing tasks based on free will accompanied by a sense of inner control. Competence involves using one's own power to be effective and achieve something, while relatedness includes interacting with other people [12]. Together, they contribute to increased motivation and general well-being. SDT separates intrinsic and extrinsic motivation, where motivation is considered a continuum from amotivation to intrinsic motivation. Intrinsic motivation inspires us to perform tasks that call upon interest and pleasure and are experienced as a reward due to the task itself being valuable, challenging, or interesting [13].

## 2. Methods

### 2.1. Study Design and Research Paradigm

The study has a descriptive and exploratory qualitative design with a phenomenological hermeneutical approach.

### 2.2. Sampling Strategy

The sampling strategy was purposive and convenient [14, 15]. The participants were selected from a list of formerly admitted patients at the rehabilitation unit. Inclusion criteria were transfemoral amputation of at least three years, because transfemoral amputation is associated with poorer physical functioning compared to transtibial amputation [2, 7]. The criterion of at least three years post amputation was chosen in order for the participants having made their own experiences over a significant amount of time [16]. Exclusion criteria were double leg amputees, known drug abuse, or mental impairment. Initial plan was 10-15 participants, but due to the corona pandemic and limited time, we recruited eight eligible participants.

### 2.3. The Sample

The sample consisted of five men and three women, with mean 70 ± 6.9 (58-77) years. The participants lived in the southern part of Norway, across four municipalities. Average time since the amputation was 11 years. Two persons in the sample were on disability benefits, and six were retired. Two of them lived alone, while the rest lived with a spouse. They had quite different level of function, from using wheelchair daily to ambulating with the prosthesis without an assistive device. The etiology for amputation was arteriosclerosis, deep vein thrombosis, infection, or trauma.

### 2.4. Data Collection and Analysis

The data collection occurred in November-January 2020-2021 through individual, semistructured interviews. An interview guide was prepared prior to the interviews (the appendix). Seven of the interviews were conducted in the participants' homes and one at the rehabilitation hospital. The interviews were audio-recorded and transcribed verbatim shortly after they occurred. The interviews lasted from 37 to 72 minutes.

Thematic analysis was used, following the six steps iteratively [17]:

1. Familiarise yourself with the data: the recorded interviews were listened to before being transcribed verbatimly, reread, and corrected while listening to the recordings
2. Generating initial codes: complete coding was chosen. Aspects of the data related to the research question were identified, and data that did not relate were not coded
3. Searching for themes: codes were actively combined to form themes that contributed to answering the research question. Subthemes were merged if similar in nature. Two main themes were defined: facilitators and barriers
4. Reviewing themes: after reevaluating the codes, transcripts were reread to ensure that themes captured the meaning of the datasets to the research question
5. Defining and naming themes: a compilation of the results was made to summarise and sort the data and to define what was unique and special to every theme and subtheme. Themes and subthemes were named
6. Producing the report: the compilation in step 5 was refined several times as part of the iterative process in a qualitative research. The report was then written as a master thesis

### 2.5. Trustworthiness

Credibility addresses whether the study measures what it intended to measure [18]. Peer debriefing is an activity to ensure this. In this study, both authors were involved throughout the process. Preconceptions have been thoroughly explored through discussion and reflection. The method has been described in detail to maintain transparency [19]. Information validation has been executed by sending the transcripts to the participants for feedback. The first author has broad experience in amputation rehabilitation, which can increase credibility.

Dependability relates to contextual conditions and has been attended to by describing the context and approach in a logical, transparent, and traceable manner [14, 19]. The transcription was undertaken shortly after the interviews, which can further enhance dependability.

Transferability [14, 20] can possibly be regarded as good to similar rehabilitation institutions. The organisation of the Norwegian health care system is an advantage to the patients because rehabilitation and prosthesis expenses are free of charge. Thus, there is probably a decreased transferability to low-income countries [21], or to countries with a personal health insurance-based system. The study does not suggest transferability for patients with disability caused by other factors than amputation, due to the technical perspective of a prosthesis.

### 2.6. Ethics

Formerly admitted patients were included in the study; hence, ethical approval was required. The Regional Ethical Committee (ID number 155207) of the University of Agder's Research Ethics Committee, Norwegian Centre for Research Data, and Soerlandet Hospital gave their approval for the study. The participants received both oral and written information prior to their consent.

The amputation environment connected to the hospital, regarding both patients and employees, is small. It was therefore crucial to secure the informant's anonymity. Only the main author knows the identity of the participants. Details that could reveal any identity have been omitted.

## 3. Results

**Figure 1:**
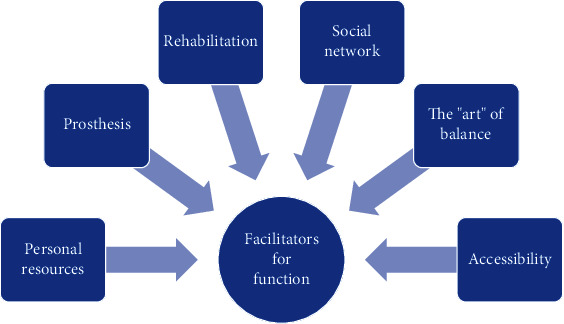
Facilitators for function.

**Figure 2:**
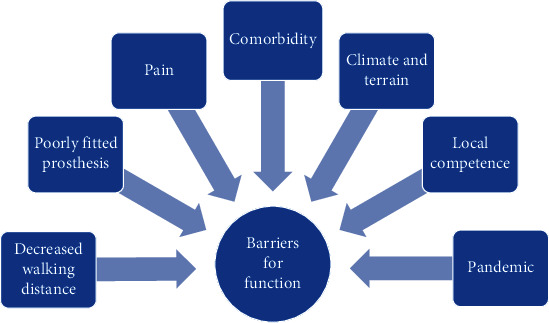
Barriers for function.

The analysis showed two main themes based on the codes in the interviews: facilitators and barriers to function. In the interpretation process, subthemes have been worked out. Themes and subthemes are shown in Figures 1 and 2.

### 3.1. Facilitators for Function

#### 3.1.1. Personal Resources

A major finding to achieve the optimal level of function was personal resources. This included multiple aspects. Several of the participants had decided to remain independent: “I decided early on when I was amputated that I would not be like- can you get me this and that? I am going to do it myself, both because of me and others.” Personal resources were also about will: “You cannot blame anyone other than yourself. If you do not want to, there is no point. Because you will have to want it- 100%,” one of the participants expressed. Another one said: “I can do a lot if I want to.” Acceptance of the situation, guts, and staying positive were other examples of personal resources that enhanced function in everyday life. The participants expressed a kind of instant acceptance and then made the best of it: “This is how it is and will be- I have accepted it.”

#### 3.1.2. Prosthesis

According to the participants, a well-fitted prosthesis was the foundation for function. In the interviews, this was expressed as “I would not have been without the prosthesis. I mean, go shopping without the leg, just in a wheelchair?” For the prosthesis to be well fitted, there must be a good collaboration with the prosthetist. In addition, the patient needed to have optimal prosthesis components which were customised to his/her level of function and activity.

#### 3.1.3. Rehabilitation

The participants highlighted the importance of rehabilitation, both primary rehabilitation shortly after amputation and readmissions down the line. In the primary stay, they learned the basics of living with a prosthesis. As time went by, new and different challenges could occur. Receiving multidisciplinary care from experienced health workers was valuable to them, described as “And that is the result when you get these three weeks stays with a physiotherapist, occupational therapist, and prosthetist. You cannot get that when you are at home, even with daily visits to the prosthetist.” Another element to rehabilitation was the opportunity to meet peers: “Yes, share some experiences, meet other amputees. That is useful.”

#### 3.1.4. Social Network

Support from social networks was important to the participants. One participant articulated “It has to do with that ‘drive' - that someone supports you 100%. I have my family. I have strong support in everybody actually.” Another one said, “my spouse supports me, and if anything were to happen, it wouldn't be long before the children came to help.” Participants experienced user organisations useful for being part of a group. Another perspective on social support was to avoid dependency on others and avoid becoming a liability. In addition, a few of the participants said they helped others, which meant they were not the only recipients of caregiving, but the relations consisted of a more mutual character.

#### 3.1.5. The “Art” of Balance

The interviews gave insight into the participants' lives and that they to a large extent manage basic daily life activities like cooking, laundrying, grocery shopping, and personal ADL. It varied whether these activities were performed with or without a prosthesis, using walking devices, or sitting in a wheelchair. Several of the participants told of more demanding activities, for instance, mowing the lawn, painting houses, carpentering, and gardening. Regardless of the activity, they pointed out that it is both time and energy consuming to perform things with the prosthesis on, and they have experienced the importance of taking breaks. One of the participants said: “I have learned to take breaks, it is almost the most important thing. Because then you can recover. You get quite tired and if you do too much, it takes days to recover. The muscles have been in, how can I put it, locked position or inactive. It takes time to rebuild them again.”

#### 3.1.6. Accessibility

Living in a home that was adapted for their disability was important to the participants, including assistive devices. It created increased possibilities for living independently. One of them put it like this: “Much is due to us having adapted everything in our house. Among many other things, we removed the thresholds. However, then all adaptations have led to me using the wheelchair indoors, because everything is accessible for me.” Half of the participants had adapted their own house, while the other half had moved to another house due to the need of adaptation that could not be done in the house they lived in prior to the amputation. Universal adaptation in their community was another side to accessibility. The participants mentioned numerous places that were not accessible for disabled persons, and they had to avoid these places.

### 3.2. Barriers for Function

#### 3.2.1. Decreased Walking Distance

One of the participants said, “I never walk from A to Z,” which can be descriptive of the limitations lower limb amputees can experience. Walking distance was one such limitation. Another participant expressed: “...walking very far. In rugged terrain. Not only on gravel, but in the forest. That is heavy. Really heavy.”

#### 3.2.2. Poorly Fitted Prosthesis

Several of the participants mentioned a poorly fitted prosthesis as a major barrier, for instance, “It has been quite painful when the prosthesis hasn't fitted. When the socket does not fit and you use it too long. That is not particularly comfortable.” Another one said that donning the prosthesis sometimes was hard and difficult. Yet another one said that the prosthetist had trouble getting the prosthesis “fit like a glove” and that the soft tissue resulted in decreased stability in the prosthesis. In addition, some participants have experienced extensive sweating in the silicone liner, to such an extent that the prosthesis fell off while walking.

#### 3.2.3. Pain

Pain was a factor several participants highlighted. As one of them said: “Clearly, there has been a lot of pain, but I have bitten my teeth together. It is painful to wear the prosthesis, but when I take it off, it is not that painful anymore. I do have energy, but I am not able to fully use my energy to do what I want because of the pain.” Another side to this subtheme was that the participants reported on sleep deprivation due to pain. Tiredness caused by pain added to the increased level of energy it took merely to ambulate with a prosthesis. An interesting feature of the interviews was that several of the participants reported increased or new phantom pain as time has passed. Expressed by one of them as “I have had this phantom pain, which is something I have not had since the amputation. The doctor gave me some pills because I did not sleep at night. I did not manage to sleep with that much pain.” While another one said: “Phantom pain and phantom sensations have increased. It is incredibly uncomfortable. Constantly bothering me. The brain needs to unplug it.” Stump pain was also reported in the sample, to such a degree that surgery could be required to solve the problem. Too much soft tissue due to a suboptimal surgery technique had resulted in pain and decreased stability for a few of the participants. Some of the participants shared how they dealt with their pain. Resting, reducing activity, adjusting the prosthesis, massaging the stump, pain medication, and diverting themselves from pain were examples of pain-reducing measures.

#### 3.2.4. Comorbidity

Comorbidity was another subtheme regarding barriers to function. In this study, this was related to cancer, diabetes, rheumatoid arthritis, or musculoskeletal disorders. One of the participants said: “I cannot stand for too long on my other knee. I simply do not have the energy for long time standing.” Another one pointed out problems like coxarthrosis and previous knee surgery, which had caused problems performing activities with the prosthesis. Weight gain can be relevant to comorbidity, even though it was not necessarily a matter of obesity. However, several of the participants mentioned that they had gained weight after the amputation due to decreased level of activity: “I became more passive. I gained quite a lot of weight over the first few years.” Age can also be relevant to comorbidity, as several of the participants reported activities and movement to be increasingly harder with age, expressed by one of them as “Clearly, age and injury, you have to be realistic in this.”

#### 3.2.5. Climate and Terrain

Most of the participants stated the Nordic climate as a barrier, along with ambulating in uneven terrain. As one put it: “In the winter. When the ground is covered in snow or ice. That is the most difficult. I feel like ‘Bambi on the ice' or trapped. That is perhaps mostly because I have a femoral amputation. It is easier if you have your own knee. I wear spikes on my shoes because I must be cautious not to fall.” Fear of falling, regardless of the icy ground, represented a barrier for the participants, expressed as “My intention was to start walking longer distances outside without crutches. However, I realised I did not dare to do that, because if I would fall, I could not get back up.” Fear of falling was also present in indoor activities: “I do not have enough balance to mop the floors. I do not feel safe enough, and I never have…. I am simply too insecure and afraid.”

#### 3.2.6. Local Competence

Several of the participants expressed coming home from primary rehabilitation as a challenging transition. They had been surrounded by a highly competent, multidisciplinary team. Coming home they were left on their own. Locally, they experienced poorer competence in challenges lower limb amputees can encounter, for example, their need of assistive devices. This implied both when they were newly amputated and as the years went by. Poorer competence regarded both receiving the needed assistive devices, but also that these devices came at the right *time*. One of the participants said: “I wish NAV (Norwegian Labour and Welfare Administration) knew a bit more. Because clearly, they make a few decisions on my behalf. I have met many closed doors. Even doors that are supposed to be open.” In addition to NAV, the participants experienced decreased competence from their general practitioner and physical therapist, which potentially constituted a barrier for development in their function.

#### 3.2.7. Pandemic

The corona pandemic resulted in social distancing, less physical activity, and less leisure activities for several of the participants. Maintaining or improving the level of function requires maintenance on a regular basis; thus, we can see that the pandemic suggests a negative impact on the participants' function. One of the participants expressed that “In times like these, I have been to very few places. A couple of times out grocery shopping, and that is it.”

## 4. Discussion

The primary objective of this study was to identify barriers and facilitators for function which lower limb amputees experience in their lives several years after amputation, from the amputee's perspective.

The purpose of prosthesis fitting is that the patient utilises the prosthesis as much as possible and achieves the highest possible level of function [8]. An important premise for this function is a well-fitted prosthesis [22]. Gailey et al. [23] claim that good socket comfort and successful use of a prosthesis are associated with the ability lower limb amputees have in performing daily activities. The individual becomes intrinsically motivated and uses the prosthesis because he/she experiences the gain of using it, cf. SDT [12]. On the other hand, if the prosthesis does not fit, the prerequisite for function is reduced. Dissatisfaction with prosthesis is by Gailey et al. [24] indicated as widespread. Between 30% and 100% denote prosthesis problems which result in discomfort or stump problems to such an extent that it influences their ability to walk [24]. Having difficulty walking will reduce their ability to perform daily activities, which in turn can reduce quality of life [24]. Decreased use of the prosthesis can result in poorer balance and less muscle strength. Several of the participants reported prosthesis issues which caused periods where they were unable to wear the prosthesis. They expressed frustration when this occurred, which can suggest that the participants considered the prosthesis to be an important and useful assistive device. However, it is easy to put the prosthesis aside if it continuously causes problems. The opportunity to perform meaningful activities can be reduced because their intrinsic motivation decreases [12].

Several of the participants did not use the prosthesis indoors but preferred wheelchair for indoor ambulation [25, 26]. These participants seemed to have experienced the prosthesis as more of a barrier than a facilitator indoors which might reduce the person's intrinsic motivation of using it. There is no positive gain despite the effort which, according to SDT, is a major prerequisite for intrinsic motivation [12]. Perhaps health workers regard indoor use of the prosthesis as more important than the person itself. It appears that the participants figured out solutions for performing activities without wearing the prosthesis, both indoor and for community dwelling. And even though we consider the prosthesis to be a prerequisite for function, a lower limb amputee can achieve a satisfying level of function even without a prosthesis. It is crucial that the patient also practices nonprosthesis function because prosthetic users sometimes cannot wear it due to stump wounds or maintenance of the prosthesis. Nonetheless, using a prosthesis will provide a more symmetrical use of the body with the health benefits this can imply [24].

About half of the participants have a microprocessor-controlled knee. They expressed an increased sense of security compared to mechanical prosthetic knees due to increased stability in the stance phase. In addition, they experienced decreased energy consumption with microprocessor-controlled knees [27]. Increased stability and decreased energy consumption can stimulate activity, hence reducing barriers. Several of the participants expressed difficulty walking in terrain. van Twillert et al. [28] claim that more advanced prosthetic knees resulted in better function in downhill walking compared to a mechanically controlled knee.

Amputees frequently suffer both stump pain and phantom pain [2, 4, 16, 25, 29, 30], but also secondary pain such as musculoskeletal disorders due to overload of the residual limb [24, 29]. The participants in the current study reported varying levels of pain, but they all had, or have had, issues with pain which constituted a barrier to function. If the pain is related to the prosthesis, a thorough analysis of gait, pain, and prosthesis can help the patient. Gailey et al. [23] say that reduced socket comfort will result in numerous visits to the prosthetist to relieve pain and decrease gait deviation. Several of the participants had an advanced prosthetic knee joint which requires weight bearing. Weight bearing on a painful stump can be hard. This can imply that the full technological potential of the prosthesis can be difficult to exploit. Hence, reducing pain can become a facilitator for function. Pain is a subjective sensation, and human beings are differently affected by pain [31]. The results in the current study suggest pain to be a barrier for function because the participants did not accomplish the activities they wished to due to pain. It is human to avoid pain, which in turn can lead to fear avoidance [32]. A multidisciplinary mapping and treatment can be helpful to minimise pain as a barrier to function [7].

One of the results in this study is the participants' strong support from their social network. An element to this is that the participants find it meaningful to be of support to others; hence, the support becomes mutual. This harmonises with other research where the authors point out that social support is achieved by caregiving [32, 33]. Social relations are important to self-image, meaning of life, and general well-being and can facilitate the continuum of motivation [12].

Being able to perform meaningful activities was expressed by most of the participants. They said it does not matter *what* you do, if you do something that you enjoy for yourself, which resonates with SDT [12]. In the context of personal resources, this can enhance function because it means orienting life in a new situation with altered prerequisites for participation and activity following a lower limb amputation.

Liu et al. [34] claim that there is a growing recognition that amputation does not always cause a negative outcome. In the current project, this was true to two of the participants. They were to a high degree involved in the decision on their own amputation. They experienced a type of relief due to excessive problems and pain prior to the amputation. Liu et al. [34] also found that psychological growth can occur as soon as six months after the amputation. Several of the participants said they early on made up their minds to cope with the amputation. From the results, it seems they have managed to find a balance in their lives. This can be a result of time passed since amputation because health indicators seem to increase as time goes by [2].

Methodological issues in this study need to be addressed. The main author, who conducted the interviews, has a previous relation to the participants as their physical therapist. This could influence the data collection in the sense that participants presented themselves with a better function than they had. On the other hand, having had a previous relation can minimise the uneven balance of power which can occur in a researcher-participant relation.

The sample size in the study was eight. Saturation [14] was achieved in the sense that significant new information ceased to occur after eight interviews. To strengthen the trustworthiness of the data from this population, more participants could have been included in the study.

## 5. Conclusion

Lower limb amputees experience barriers in their everyday life, but they also develop strategies to cope with their disability. Intrinsic motivation for prosthetic use depends on whether the amputee experiences the prosthesis as a useful assistive device or not.

Further research suggests executing a similar project with transtibial or bilateral amputees.

### 5.1. Clinical Implications for Rehabilitation

- Keeping a stable, sound body weight is important to make the prosthesis fit optimally, as well as the benefits this will have on general health. Amputees seem to have an increased risk of gaining weight; hence, extended use of nutritionist post discharge can supply the patient with necessary nutritional support
- Although the participants in this project currently did not express psychological issues, it can be psychologically challenging living as an amputee. Structural psychological mapping can monitor and/or suggest treatment and follow-ups. This is especially important when the patient is discharged from primary rehabilitation and returns to their local community
- With time, the amputee's level of function varies. It is important that follow-ups in the clinical pathway stretch over a significant period because the participants express interactions with a multidisciplinary rehabilitation unit as useful and necessary
- The pandemic has accelerated use of digital consultations. Extended use of digital consultation can provide better accessibility to specialised health care workers, decrease travel time to/from consultations, and more easily include local health care workers

## Data Availability

Due to the nature of this research, participants of this study did not agree for their data to be shared publicly, so supporting data is not available.

## Appendix

### A. Interview Guide

Informal chat (5 minutes)

Information (5-10 minutes):

- Theme, background, and purpose of the interview
- Explain how the interview will be used
- The researchers' duty of confidentiality and the participants' anonymity
- About recording. Receive consent
- Questions from the participant
- Start recorder

Transitional questions (5-10 minutes):

- How long have you been amputated?
- How much do you use your prosthesis in a day?
- How close to your preferred level of function are you?
- What is your understanding of the term level of function?

Key questions (45 minutes):

- Can you describe what you do in a regular day?
- Which activities or movements are easy for you to do?
- What do you succeed in and why?
- In your experience, what can be difficult to perform?
- What do you think helps you to perform activities or tasks?
- In your everyday life, what do you think makes activities or tasks more difficult or stops you from doing things?
- Who is important to you to perform activities or tasks?
- How do you experience being an amputee?

Relevant follow-up questions:

- That is interesting, can you explain closer?
- Could you give me an example?
- Could you tell me more about that?
- Is there anything else you want to tell?

Summary of the interview (5-10 minutes):

- Have I understood you correctly? Is this a correct summary of our conversation?
- Is there anything else you would like to add or elaborate on?
- Stop recorder

We thank you for the participation. Information about professional help in case of any psychological reactions was given to each participant.
